# *α*-Glucosidase Inhibitory Phytochemical Components of Chinese Endemic Plant *Whitfordiodendron filipes* var. *tomentosum*

**DOI:** 10.3390/plants13050692

**Published:** 2024-02-29

**Authors:** Jun-Kun Chen, Zeng-Yue Ge, Xiao-Wen Liao, Jun Xue, Lei Wu, Lin-Fu Liang

**Affiliations:** 1 College of Materials Science and Engineering, Central South University of Forestry and Technology, 498 South Shaoshan Road, Changsha 410004, China; 2 College of Forestry, Central South University of Forestry and Technology, 498 South Shaoshan Road, Changsha 410004, China

**Keywords:** triterpene, steroid, hypoglycemic bioactivity, molecular docking

## Abstract

*Whitfordiodendron filipes* var. *tomentosum* is an endemic plant in China. There have been no chemical or pharmacological studies of this plant reported before. In the current research, eight triterpenes and two steroids were obtained. Their structures were established by the analysis of NMR data and comparison with those reported in the literature. These ten structurally diverse compounds comprised five distinct carbon frameworks with different functionalities. The chemotaxonomic significance of these secondary metabolites was discussed, disclosing the common components between the variant *W. filipes* var. *tomentosum* and the species *W. filipe*. Evaluation of *α*-glucosidase inhibitory activities of these isolates disclosed that compounds **1**, **2**, **4**, and **6** exhibited significant *α*-glucosidase inhibitory activities (IC_50_ = 16.6–19.2 μM), which were close in value to the positive control acarbose (IC_50_ = 11.5 μM). Moreover, the binding modes between the biologically active compounds **1**, **2**, **4**, and **6** and the *α*-glucosidase protein were preliminarily studied using molecular docking. This study not only showed the chemical and biological profile of the plant *W. filipes* var. *tomentosum* but also revealed that these components could be developed as hypoglycemic lead compounds.

## 1. Introduction

It is well known that traditional Chinese medicine (TCM) plays a key role in the treatment of various diseases including cancers [1] and malaria [2] in China and other countries. Herbal drugs are frequently utilized biological materials in TCM. For instance, the herbs of the genus *Artemisia* have been used as the main component of the Chinese prescription for treating malaria [2] because these plants contain an abundance of artemisinin and its derivatives. Prof. Youyou Tu, who made a great contribution to the exploration and utilization of artemisinin, was awarded the Nobel Prize in Physiology or Medicine in 2015 [3]. A vast library of studies demonstrates the benefits of ginsengs in cancer treatments. Ginsenoside Rg3, which was a triterpene isolated from red ginseng (Ginseng Radix et Rhizoma Rubra), exhibited remarkable antitumor effects against various types of cancers, including lung, liver, and breast cancer. This compound has been developed as a single-compound TCM drug, the Shenyi capsule, which was approved in 2003 in China [1]. Polyprenylated xanthones of gamboge resin produced by various *Garcinia* species, including *Garcinia hanburyi*, showed outstanding cytotoxic activity against various malignant tumors. Gambogic acid, one of the active components of resin, was approved for clinical trials as a class 1 new drug in 2004 by the Chinese Food and Drug Administration [1]. Continuous investigations revealed that many plants can produce a diverse array of bioactive compounds, some of which are also used as drugs, such as paclitaxel, morphine, ephedrine, and ferulic acid [4,5,6]. Inspired by these exciting works, more and more natural product researchers [7] and synthetic chemists [8] have shown great interest in this field.

The genus *Whitfordiodendron* comprises ca. nine species distributed mainly in Asian and Oceanian countries including China, the Philippines, Indonesia, Malaysia, and Australia [9]. Specifically, one species of this genus, *Whitfordiodendron filipes*, and its variant, *Whitfordiodendron filipes* var. *tomentosum*, are endemic plants in southwestern China. They were recorded as medicinal plants with diverse pharmacological functions such as anti-diabetes-related effects [10]. A literature survey revealed that extracts of the plant *W. filipes* contained alkaloids [10,11,12,13], flavonoids [10,11,12,13,14], terpenes [10,13,14], steroids [13,14], fatty acids, esters [13,14], and other miscellaneous lipids [10,14]. Although different kinds of secondary metabolites of this species have been reported, only the antioxidant effect of the extract of *W. filipes* was assessed [13]. It might be worth pointing out that there have been no phytochemical studies on *W. filipes* var. *tomentosum* reported before.

*α*-Glucosidase is a key enzyme that hydrolyzes carbohydrates to *α*-glucose, which is absorbed in the small intestine [15]. The inhibition of *α*-glucosidase could retard the digestion of glucose and downgrade the postprandial blood glucose levels [16]. As a result, *α*-glucosidase inhibitors, such as acarbose, have been developed as effective drugs in the treatment of diabetes [17]. In recent years, continuous efforts have been made to search for natural potent *α*-glucosidase inhibitors as lead compounds against diabetes [18].

With this aim and in the course of our continuing research on bioactive secondary metabolites from TCM [19,20,21], we collected the Chinese endemic plant *W. filipes* var. *tomentosum*. Chemical investigation of the ethanol extract of this plant led to the isolation of ten structurally diverse compounds. Moreover, the potential *α*-glucosidase inhibitory activities of these isolates were evaluated.

## 2. Results and Discussion

### 2.1. Phytochemical Components

**Figure 1:**
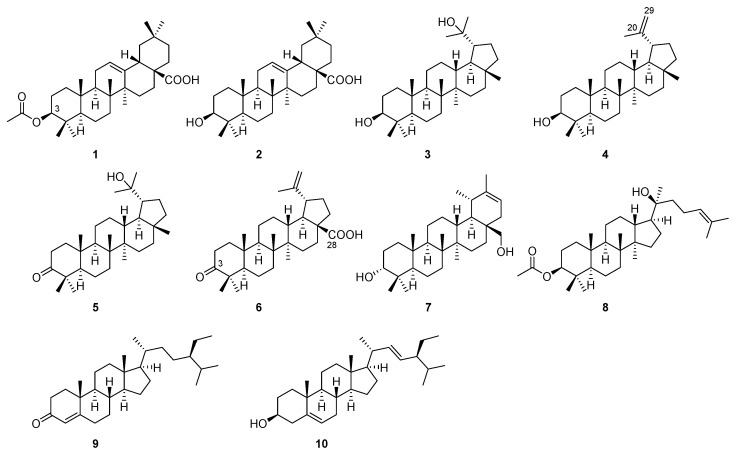
Chemical structures of compounds **1**–**10**.

The dried caulis of *W. filipes* var. *tomentosum* was pulverized and extracted with ethanol three times. Then, the ethanol extract was successively partitioned with petroleum ether and ethyl acetate. From these two above-mentioned extracts, ten known compounds were obtained after multiple rounds of chromatography. Their structures are shown in Figure 1, which were identified by the analysis of NMR data and comparison with those reported in the literature.

Compound **1** was isolated as a white amorphous powder. Its ^1^H and ^13^C NMR spectra displayed the signals attributed to one carboxyl carbon [*δ*_C_ 184.1 (C-28)], one olefinic bond [*δ*_H_ 5.27 (1H, t, *J* = 3.5 Hz, H-12); *δ*_C_ 122.6 (C-12), 143.6 (C-13)], one oxygenated methine [*δ*_H_ 4.49 (1H, t, *J* = 8.0 Hz, H-3); *δ*_C_ 80.9 (C-3)], one acetyl [*δ*_H_ 2.04 (3H, s, CO*CH_3_*); *δ*_C_ 21.3 (CO*CH_3_*), 171.0 (*CO*CH_3_)], and seven singlet methyls [*δ*_H_ 1.13 (3H, s, H_3_-27), 0.94 (3H, s, H_3_-25), 0.93 (3H, s, H_3_-30), 0.90 (3H, s, H_3_-29), 0.85 (3H, s, H_3_-24), 0.83 (3H, s, H_3_-23), 0.75 (3H, s, H_3_-26); *δ*_C_ 28.1 (C-23), 16.7 (C-24), 15.4 (C-25), 17.2 (C-26), 25.9 (C-27), 33.1 (C-29), 23.6 (C-30)]. The 32 carbon signals indicated this compound was a triterpene with an acetyl group. A literature survey revealed that the overall ^1^H and ^13^C NMR spectra data of **1** were identical to those reported for the oleane-type triterpene 3-acetyloleanolic acid [22]. Hereto, the structure of compound **1** was determined as depicted in Figure 1.

Similar to compound **1**, the ^1^H and ^13^C NMR spectra of **2** also showed the signals for one carboxyl carbon [*δ*_C_ 183.2 (C-28)], one olefinic bond [*δ*_H_ 5.27 (1H, t, *J* = 3.5 Hz, H-12); *δ*_C_ 122.4 (C-12), 143.0 (C-13)], one oxygenated methine [*δ*_H_ 3.21 (1H, dd, *J* = 10.0, 4.5 Hz, H-3); *δ*_C_ 79.3 (C-3)], and seven singlet methyls [*δ*_H_ 1.11 (3H, s, H_3_-27), 0.99 (3H, s, H_3_-23), 0.94 (3H, s, H_3_-30), 0.93 (3H, s, H_3_-25), 0.92 (3H, s, H_3_-29), 0.76 (3H, s, H_3_-24), 0.73 (3H, s, H_3_-26); *δ*_C_ 28.0 (C-23), 15.6 (C-24), 15.3 (C-25), 17.2 (C-26), 26.2 (C-27), 33.1 (C-29), 23.8 (C-30)]. Although their ^1^H and ^13^C NMR data were almost the same, the latter did not have NMR signals for the acetyl group. The lack of acetyl functionality was supported by the significant upfield-shifted chemical shifts for the methine CH-3 [**2**: *δ*_H_ 3.21 (1H, dd, *J* = 10.0, 4.5 Hz, H-3); *δ*_C_ 79.3 (C-3) vs. **1**: *δ*_H_ 4.49 (1H, t, *J* = 8.0 Hz, H-3); *δ*_C_ 80.9 (C-3)]. Consequently, compound **2** was assigned as oleanolic acid, which was confirmed by the superposable NMR data reported in the literature [23].

In the ^1^H and ^13^C NMR spectra of **3**, one oxygenated methine [*δ*_H_ 3.14 (1H, dd, *J* = 11.7, 5.5 Hz, H-3); *δ*_C_ 79.6 (C-3)], one oxygenated quaternary carbon [*δ*_C_ 73.0 (C-20)], and eight singlet methyls [*δ*_H_ 1.20 (3H, s, H_3_-29), 1.10 (3H, s, H_3_-30), 1.04 (3H, s, H_3_-25), 0.96 (3H, s, H_3_-26), 0.94 (3H, s, H_3_-27), 0.87 (3H, s, H_3_-24), 0.79 (3H, s, H_3_-23), 0.73 (3H, s, H_3_-28); *δ*_C_ 28.7 (C-23), 15.6 (C-24), 16.5 (C-25), 16.9 (C-26), 15.6 (C-27), 18.6 (C-28), 24.1 (C-29), 31.2 (C-30)] were observed. Since the number of carbon signals was 30, this compound was a triterpene too. Finally, based on the comparison with the data in the literature [24], this compound was identified as a lupane-type triterpene monogynol A.

The ^1^H and ^13^C NMR spectra of **4** revealed the presence of one terminal olefinic bond [*δ*_H_ 4.68 (1H, s, H_a_-20), 4.55 (1H, s, H_b_-20); *δ*_C_ 151.0 (C-20), 109.3 (C-29)], one oxygenated methine [*δ*_H_ 3.20 (1H, m, H-3); *δ*_C_ 79.1 (C-3)], one allylic methyl [*δ*_H_ 1.67 (3H, s, H_3_-30); *δ*_C_ 21.0 (C-30)], and six other singlet methyls [*δ*_H_ 1.02 (3H, s, H_3_-27), 0.95 (3H, s, H_3_-24), 0.86 (3H, s, H_3_-25), 0.82 (3H, s, H_3_-23), 0.78 (3H, s, H_3_-28), 0.75 (3H, s, H_3_-26); *δ*_C_ 30.0 (C-23), 16.1 (C-24), 18.1 (C-25), 14.7 (C-27), 25.2 (C-28), 16.2 (C-26)]. As observed, its ^1^H and ^13^C NMR data closely resembled those of **3**. However, they differed by the replacement of propan-2-ol in **3** [*δ*_H_ 1.20 (3H, s, H_3_-29), 1.10 (3H, s, H_3_-30); *δ*_C_ 73.0 (C-20), 24.1 (C-29), 31.2 (C-30)] with prop-2-ene in **4** [*δ*_H_ 4.68 (1H, s, H_a_-20), 4.55 (1H, s, H_b_-20), 1.67 (3H, s, H_3_-30); *δ*_C_ 151.0 (C-20), 109.3 (C-29), 21.0 (C-30)]. Based on the above-mentioned analysis, the structure of **4** was proposed as lupeol, which was supported by the identical NMR data listed in the literature [13].

Interestingly, the ^1^H and ^13^C NMR spectra of **5** exhibited close similarity to those of **3**, including one oxygenated quaternary carbon [*δ*_C_ 73.6 (C-20)] and eight singlet methyls [*δ*_H_ 1.23 (3H, s, H_3_-29), 1.12 (3H, s, H_3_-30), 1.09 (3H, s, H_3_-25), 1.07 (3H, s, H_3_-26), 1.03 (3H, s, H_3_-27), 0.97 (3H, s, H_3_-24), 0.94 (3H, s, H_3_-23), 0.82 (3H, s, H_3_-28); *δ*_C_ 26.8 (C-23), 21.2 (C-24), 16.2 (C-25, C-26), 14.9 (C-27), 19.4 (C-28), 31.8 (C-29), 24.9 (C-30)]. Their difference was in the appearance of a carbonyl [*δ*_C_ 218.3 (C-3)] in **5** instead of the oxygenated methine [*δ*_H_ 3.14 (1H, dd, *J* = 11.7, 5.5 Hz, H-3); *δ*_C_ 79.6 (C-3)] in **3**. Accordingly, compound **5** was identified as 20-hydroxy-lupan-3-one, whose data matched well with those recorded in the literature [25].

As indicated by the ^1^H and ^13^C NMR data, compound **6** had one carbonyl carbon [*δ*_C_ 218.5 (C-3)], one carboxyl carbon [*δ*_C_ 180.0 (C-28)], one terminal olefinic bond [*δ*_H_ 4.71 (1H, s, H_a_-29), 4.59 (1H, s, H_b_-29); *δ*_C_ 152.0 (C-20), 110.2 (C-29)], one allylic methyl [*δ*_H_ 1.70 (3H, s, H_3_-30); *δ*_C_ 19.5 (C-30)], and five other singlet methyls [*δ*_H_ 1.44 (3H, s, H_3_-23), 1.06 (3H, s, H_3_-24), 1.02 (3H, s, H_3_-26), 1.01 (3H, s, H_3_-27), 0.95 (3H, s, H_3_-25); *δ*_C_ 27.2 (C-23), 21.4 (C-24), 16.5 (C-25), 16.4 (C-26), 15.0 (C-27)]. These characteristic NMR signals revealed the close structural relationship between compounds **6** and **4**, except that the oxidation occurred at C-3 [**6**: *δ*_C_ 218.5 (C-3) vs. **4**: *δ*_C_ 79.1 (C-3)] and C-28 [**6**: *δ*_C_ 180.0 (C-28) vs. **4**: *δ*_C_ 25.2 (C-28)]. Through comparison with the data in the literature [26], this compound was identified as betulonic acid.

The ^1^H and ^13^C NMR spectra of compound **7** displayed the signals attributed to one olefinic bond [*δ*_H_ 5.30 (1H, d, *J* = 7.0 Hz, H-21); *δ*_C_ 141.2 (C-20), 118.0 (C-21)], one oxygenated methine [*δ*_H_ 3.40 (1H, t, *J* = 2.8 Hz, H-3); *δ*_C_ 76.4 (C-3)], one hydroxymethyl [*δ*_H_ 3.67 (1H, d, *J* = 11.0 Hz, H_a_-28), 3.48 (1H, d, *J* = 11.0 Hz, H_b_-28); *δ*_C_ 60.4 (C-28)], one allylic methyl [*δ*_H_ 1.65 (3H, s, H_3_-30); *δ*_C_ 21.5 (C-30)], one doublet methyl [*δ*_H_ 1.00 (3H, d, *J* = 6.5 Hz, H_3_-29); *δ*_C_ 22.3 (C-29)], and five other singlet methyls [*δ*_H_ 1.03 (3H, s, H_3_-25), 0.99 (3H, s, H_3_-24), 0.94 (3H, s, H_3_-27), 0.86 (3H, s, H_3_-23), 0.83 (3H, s, H_3_-26); *δ*_C_ 28.4 (C-23), 23.1 (C-24), 16.2 (C-25), 16.2 (C-26), 15.1 (C-27)]. These characteristic NMR signals were reminiscent of a possible taraxastane carbon framework. Through the extensive literature survey, compound **7** was identified as 20-taraxastene-3*R*,28-diol based on the well-matched data [27].

As shown in the ^1^H and ^13^C NMR spectra of compound **8**, the presence of one olefinic bond [*δ*_H_ 5.12 (1H, t, *J* = 6.5 Hz, H-24); *δ*_C_ 124.9 (C-24), 131.7 (C-25)], one oxygenated methine [*δ*_H_ 4.48 (1H, dd, *J* = 10.6, 5.7 Hz, H-3); *δ*_C_ 81.1 (C-3)], one oxygenated quaternary carbon [*δ*_C_ 75.5 (C-20)], one acetyl [*δ*_H_ 2.04 (3H, s, CO*CH_3_*); *δ*_C_ 21.4 (CO*CH_3_*), 171.1 (*CO*CH_3_)], two allylic methyls [*δ*_H_ 1.69 (3H, s, H_3_-26), 1.62 (3H, s, H_3_-27); *δ*_C_ 25.9 (C-26), 17.8 (C-27)], and six other singlet methyls [*δ*_H_ 1.14 (3H, s, H_3_-21), 0.96 (3H, s, H_3_-18), 0.87 (3H, s, H_3_-28), 0.85 (9H, s, H_3_-19, H_3_-29, H_3_-30); *δ*_C_ 15.7 (C-18), 16.6 (C-19), 25.6 (C-21), 28.1 (C-28), 16.6 (C-29), 16.4 (C-30)] was recognized. The literature survey revealed that the overall ^1^H and ^13^C NMR spectra data of **8** were identical to those reported for the dammarane-type triterpene dammar-24-ene-3*β*-acetoxy-20*S*-ol (**8**) [28]. Consequently, the structure of compound **8** was assigned as shown in Figure 1.

Compound **9** was isolated as a white amorphous powder. Its ^1^H and ^13^C NMR spectra displayed the signals attributed to one carbonyl carbon [*δ*_C_ 199.6 (C-3)], one olefinic bond [*δ*_H_ 5.70 (1H, s, H-4); *δ*_C_ 123.8 (C-4), 171.7 (C-5)], and an array of methyls including one triplet [*δ*_H_ 0.82 (3H, t, *J* = 7.4 Hz, H_3_-29); *δ*_C_ 12.1 (C-29)], three double methyls [*δ*_H_ 0.99 (3H, d, *J* = 6.6 Hz, H_3_-21), 0.87 (3H, d, *J* = 7.0 Hz, H_3_-26), 0.79 (3H, d, *J* =7. 0 Hz, H_3_-27); *δ*_C_ 36.2 (C-21), 21.1 (C-26), 21.1 (C-27)], and two singlet methyls [*δ*_H_ 1.16 (3H, s, H_3_-19), 0.70 (3H, s, H_3_-18); *δ*_C_ 12.0 (C-18), 19.1 (C-19)]. The characteristic NMR data suggested this compound was likely a steroid. Comparison with the data recorded in the literature revealed that the overall ^1^H and ^13^C NMR spectra data of compound **9** were identical to those for (20*R*)-24-ethylcholest-4-en-3-one [29]. Thus, the structure of compound **9** was determined as shown in Figure 1.

The ^1^H and ^13^C NMR spectra of compound **10** indicated similar structural features to those of **9**, except for the upfield-shifted olefinic bond [*δ*_H_ 5.70 (1H, s, H-4); *δ*_C_ 123.8 (C-4), 171.7 (C-5)], along with an oxygenated methine [*δ*_H_ 3.51 (1H, m, H-3); *δ*_C_ 71.5 (C-3)] and an additional 1,2-disubstitued olefinic bond [*δ*_H_ 5.14 (1H, dd, *J* = 15.2, 8.6 Hz, H-22), 5.00 (1H, dd, *J* = 15.5, 8.6 Hz, H-23); *δ*_C_ 139.0 (C-22), 129.2 (C-23)]. These differences revealed the nuclei of **10** were reduced, whereas the side chain was dehydrogenated. An extensive literature survey guided by the above-mentioned analysis finally identified this compound as stigmasterol [13].

### 2.2. Chemotaxonomic Significance

Worth mentioning was that there had been no chemical study reported for the plant *W. filipes* var. *tomentosum* before. Therefore, all ten compounds could be regarded as chemotaxonomic markers for this variant. In the present study, eight triterpenes (**1**–**8**) and two steroids (**9** and **10**) were discovered from the title plant. Structurally, these eight triterpenes were further categorized into four groups based on their different types of skeletons: two oleanes (**1** and **2**), four lupanes (**3**–**6**), one taraxastane (**7**), and one dammarane (**8**). This finding disclosed the diverse carbon frameworks of the triterpenoidal secondary metabolites derived from the plant *W. filipes* var. *tomentosum*. Moreover, the discovery of triterpenes and steroids enriched the chemical diversities of the species *W. filipes*. It was found that the triterpene lupeol (**4**) and the steroid stigmasterol (**10**) were also obtained from the plant *W. filipes* [13], which might be evidence of the close taxonomic relationship between the species and its variant from the chemical perspective.

### 2.3. α-Glucosidase Inhibitory Activity

All the isolated compounds from the title plant were evaluated for their α-glucosidase inhibitory activities (Figure S1, Table S1), cytotoxicity against human lung adenocarcinoma cells A549 and human breast cancer cells MCF-7, and inhibitory effects against demethylases ALKBH3 and FTO/ALKBH5. However, none of them showed potent bioactivities in the preliminary experiments except in the α-glucosidase inhibitory bioassay. The results of the bioassay (Table 1) revealed that compounds **1**, **2**, **4**, and **6** exhibited significant inhibitory activities against α-glucosidase, whereas the other compounds **3**, **5,** and **7**–**10** were judged as inactive. The IC_50_ values of the four compounds against α-glucosidase were 16.8, 16.6, 19.2, and 17.2 μM, respectively, which were close in value to the positive control acarbose (IC_50_ = 11.5 μM). This bioassay disclosed that these chemical constituents were responsible for the hypoglycemic effect of the title plant and also indicated these components could be developed as potent hypoglycemic lead compounds.

The literature survey revealed that there were α-glucosidase inhibitory bioassays reported for compounds **1**, **2**, **4**, and **6**. Chemical investigation of the dried, powdered trunks of *Coffea canephora* led to the isolation of compounds **1** and **2**, which exhibited α-glucosidase inhibitory activities with IC_50_ values of 146.9 ± 1.2 and 202.7.0 ± 0.9 μM, respectively [16]. Compound **4** was obtained from the dried stems and leaves of *Sabia parviflora*, which showed inhibitory against α-glucosidase with an IC_50_ value of 452.2 ± 5.3 μM [30]. Compound **6** was one of the chemical constituents of the leaves of *Buddleja saligna*, which displayed α-glucosidase inhibitory activity with an IC_50_ value of 60.11 ± 0.00 μM [31]. The results described in the bibliography corroborated the effects of these four isolates, although the IC_50_ values were different from our results.

### 2.4. Strucuture–Activity Relationship Analysis

Analysis of the structure–activity relationship of oleane-type triterpenes **1** and **2** revealed that the acetylation at C-3 almost had no impact on the α-glucosidase inhibitory activity. Considering the loss of α-glucosidase inhibitory activity for lupane-type triterpene **3** and the close structural similarity between compounds **3** and **4**, the terminal olefinic bond Δ^20(29)^ of the lupane skeleton might play a crucial role in the α-glucosidase inhibition. Of more interest was the recognition of the possible synergistic effect of 3-oxo and 28-COOH groups, which was indicated by the slight increase in inhibitory activity against α-glucosidase for **6** with respect to that of **4**. Further study on the more detailed structure–activity relationship and the mechanism will be conducted along with the chemical modifications in the future.

### 2.5. In Silico Study of Binding Modes

**Figure 2:**
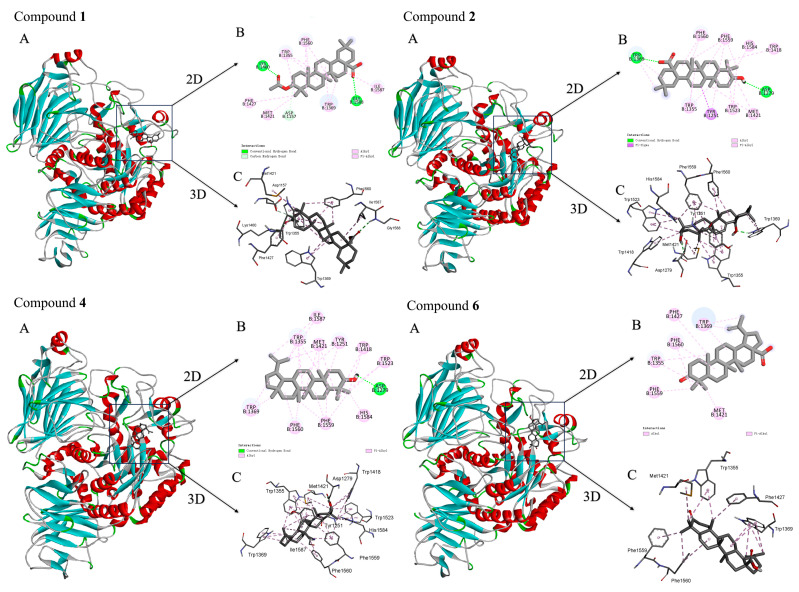
The binding modes of compounds **1**, **2**, **4**, and **6** with *α*-glucosidase: A represents the overview of binding mode; B represents the two-dimensional diagram of interactions; C represents the three-dimensional diagram of the interactions.

In this study, molecular docking studies were performed to decipher the binding modes of the bioactive compounds **1**, **2**, **4**, and **6** and the α-glucosidase protein (PDB ID: 3TOP). The images of docked complexes and molecular surfaces, as well as 2D and 3D interactive plots for compounds with the α-glucosidase protein, were shown in Figure 2.

As illustrated, compound **1** formed a hydrogen bond between its acetyl group and Lys1460, and a hydrogen bond was formed between its carboxyl group and Gly1588. Additionally, it established hydrogen bonds between its tertiary carbon and Asp1157, which were both located in the active site (the upper left part of Figure 2). Furthermore, compound **1** exhibited alkyl interactions with the amino acid residues Met1421 and Ile1587 within the receptor protein cavity and also formed π-alkyl stacking interactions with the amino acid residues Phe1427, Trp1369, Trp1355, and Phe1560. As for compound **2**, its carboxyl and hydroxyl groups formed hydrogen bonds with Trp1369 and Asp1279 (the upper right part of Figure 2).

The interaction between *α*-glucosidase crystals and compound **4** involved not only hydrogen bonds but also alkyl and π-alkyl interactions. The π-σ interactions with the amino acid residue Tyr1251, as well as π-alkyl stacking interactions with Trp1355, Trp1523, Trp1418, His1584, Phe1559, and Phe1560, were observed. It also formed a π-alkyl interaction with the amino acid residue Met1421 (the lower left part of Figure 2), while for compound **6**, its hydroxyl group formed a hydrogen bond with Asp1279. Furthermore, **6** also engaged in an alkyl interaction with both Ile1587 and Met1421 and formed π-alkyl stacking interactions with Trp1355, Tyr1251, Trp1418, Trp1523, His1584, Phe1559, Phe1560, and Trp1369 (the lower right part of Figure 2).

## 3. Materials and Methods

### 3.1. General Experimental Procedures

NMR spectra were measured on a Bruker DRX-400, Bruker DRX-500, or Bruker DRX-600 spectrometer (Bruker Biospin AG, Fällanden, Germany). Commercial silica gel (200–300 and 300–400 mesh, Qingdao Haiyang Chemical Group Co., Ltd., Qingdao, China) and Sephadex LH-20 gel (Amersham Biosciences, Amersham, UK) were used for column chromatography, and precoated silica gel plates (GF-254, Yan Tai Zi Fu Chemical Group Co., Yantai, China) were used for analytical TLC. All solvents used for column chromatography were of analytical grade (Shanghai Chemical Reagents Co., Ltd., Shanghai, China).

### 3.2. Plant Material

The plant samples of *W. filipes* var. *tomentosum* were collected from Napo County, Guangxi Autonomous Region, China, in 2018 and authenticated by Dr. L. Wu of Central South University of Forestry and Technology (CSUFT). A botanical specimen (P-2018-GXNP1) was deposited at the Laboratory of Natural Product Chemistry, CSUFT.

### 3.3. Extraction and Isolation

The dried and powdered caulis of *W. filipes* var. *tomentosum* (1.15 kg) were extracted by maceration with ethanol (3 × 7 days) at room temperature. The ethanol extract was evaporated under reduced pressure to give a dark residue, which was then suspended in water for liquid–liquid extraction and successively extracted with petroleum ether and ethyl acetate to obtain their corresponding fractions.

The ethyl acetate extract was subjected to silica gel (200–300 mesh, Qingdao Haiyang Chemical Group Co., Ltd., Qingdao, China) column chromatography (CC) by eluting with petroleum ether (P)/ethyl acetate (E) solvent system at a ratio of 100:0 to 0:100 to give eighteen fractions (Fr. E1–E18). Compound **1** (15.2 mg) was obtained from Fr. E3 following a two-stage separation beginning with silica gel CC (300–400 mesh, Qingdao Haiyang Chemical Group Co., Ltd., Qingdao, China) eluted with petroleum ether (P)/ethyl acetate (E) (50:1, 30:1, 10:1), followed by Sephadex LH-20 gel (Amersham Biosciences, Amersham, UK) eluted with P/dichromethane (D)/methanol (M) (2:1:1). Similarly, compound **2** (20.4 mg) was isolated from Fr. E6 by chromatographing over silica gel with P/E (50:1, 30:1, 10:1) and then Sephadex LH-20 with P/D/M (2:1:1). Fr. E10 was separated by repeated silica gel CC (30:1, 10:1, 5:1) to afford compound **3** (10.3 mg). Fr. E16 was divided into seven subfractions (Fr. E16A–E16G) by silica gel CC eluted with P/E (30:1, 15:1, 5:1). Fr. E16D was further purified by Si gel CC eluted with P/E (15:1, 10:1, 5:1) to give compounds **4** (382.4 mg) and **9** (95.4 mg). By repeated silica gel CC with P/E (15:1, 10:1, 5:1), Fr. E16F yielded compounds **5** (11.6 mg) and **7** (13.2 mg). Fr. E16G was purified by silica gel CC with D/M (18:1) followed by Sephadex LH-20 with P/D/M (2:1:1) to afford compound **6** (22.1 mg).

The petroleum ether extract was separated by silica gel column chromatography and eluted with gradient P/D mixture (20:1, 10:1, 3:1), yielding eight fractions (Fr. P1–P8). Fr. P4 was purified by silica gel column chromatography (P/D = 15:1, 10:1) to give compound **8** (20.8 mg). Similarly, compound **10** (20.6 mg) was afforded from Fr. P6 by multiple rounds of silica gel column chromatography eluted with D/M (P/D = 12:1, 6:1).

**Figure 3:**
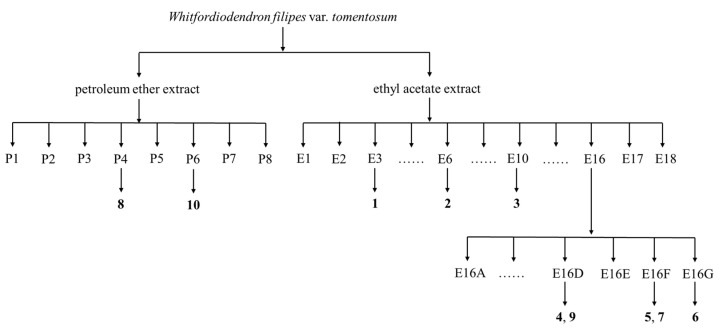
The separation and purification process of compounds **1**–**10**.

The above-mentioned separation and purification process is shown in Figure 3.

### 3.4. Characteristic ^1^H and ^13^C NMR Spectral Data

Compound **1**: ^1^H NMR (CDCl_3_, 500 MHz): *δ*_H_ 5.27 (1H, t, *J* = 3.5 Hz, H-12), 4.49 (1H, t, *J* = 8.0 Hz, H-3), 2.81 (1H, dd, *J* = 14.3, 4.0 Hz, H-18), 2.04 (3H, s, CO*CH_3_*), 1.13 (3H, s, H_3_-27), 0.94 (3H, s, H_3_-25), 0.93 (3H, s, H_3_-30), 0.90 (3H, s, H_3_-29), 0.85 (3H, s, H_3_-24), 0.83 (3H, s, H_3_-23), 0.75 (3H, s, H_3_-26); ^13^C NMR (CDCl_3_, 125 MHz): *δ*_C_ 38.1 (C-1), 23.6 (C-2), 80.9 (C-3), 37.7 (C-4), 55.3 (C-5), 18.2 (C-6), 32.5 (C-7), 39.3 (C-8), 47.6 (C-9), 37.0 (C-10), 22.9 (C-11), 122.6 (C-12), 143.6 (C-13), 41.6 (C-14), 28.1 (C-15), 23.4 (C-16), 46.6 (C-17), 39.3 (C-18), 45.9 (C-19), 30.7 (C-20), 33.8 (C-21), 32.5 (C-22), 28.1 (C-23), 16.7 (C-24), 15.4 (C-25), 17.2 (C-26), 25.9 (C-27), 184.1 (C-28), 33.1 (C-29), 23.6 (C-30), 21.3 (CO*CH_3_*), 171.0 (*CO*CH_3_).

Compound **2**: ^1^H NMR (CDCl_3_, 400 MHz): *δ*_H_ 5.27 (1H, t, *J* = 3.5 Hz, H-12), 3.21 (1H, dd, *J* = 10.0, 4.5 Hz, H-3), 2.79 (1H, dd, *J* =13.3, 4.2 Hz, H-18), 1.11 (3H, s, H_3_-27), 0.99 (3H, s, H_3_-23), 0.94 (3H, s, H_3_-30), 0.93 (3H, s, H_3_-25), 0.92 (3H, s, H_3_-29), 0.76 (3H, s, H_3_-24), 0.73 (3H, s, H_3_-26); ^13^C NMR (CDCl_3_, 150 MHz): *δ*_C_ 38.3 (C-1), 26.9 (C-2), 79.3 (C-3), 39.3 (C-4), 55.4 (C-5), 18.6 (C-6), 32.9 (C-7), 39.2 (C-8), 47.8 (C-9), 37.4 (C-10), 23.4 (C-11), 122.4 (C-12), 143.0 (C-13), 41.1 (C-14), 27.6 (C-15), 23.5 (C-16), 46.7 (C-17), 41.0 (C-18), 46.2 (C-19), 30.9 (C-20), 33.7 (C-21), 32.4 (C-22), 28.0 (C-23), 15.6 (C-24), 15.3 (C-25), 17.2 (C-26), 26.2 (C-27), 183.2 (C-28), 33.1 (C-29), 23.8 (C-30).

Compound **3**: ^1^H NMR (CDCl_3_, 400 MHz): *δ*_H_ 3.14 (1H, dd, *J* = 11.7, 5.5 Hz, H-3), 1.20 (3H, s, H_3_-29), 1.10 (3H, s, H_3_-30), 1.04 (3H, s, H_3_-25), 0.96 (3H, s, H_3_-26), 0.94 (3H, s, H_3_-27), 0.87 (3H, s, H_3_-24), 0.79 (3H, s, H_3_-23), 0.73 (3H, s, H_3_-28); ^13^C NMR (CDCl_3_, 125 MHz): *δ*_C_ 38.4 (C-1), 27.1 (C-2), 79.6 (C-3), 39.4 (C-4), 55.1 (C-5), 18.3 (C-6), 34.3 (C-7), 40.6 (C-8), 50.8 (C-9), 37.7 (C-10), 21.2 (C-11), 28.4 (C-12), 37.2 (C-13), 44.3 (C-14), 27.2 (C-15), 35.1 (C-16), 42.7 (C-17), 47.0 (C-18), 49.2 (C-19), 73.0 (C-20), 29.8 (C-21), 39.8 (C-22), 28.7 (C-23), 15.6 (C-24), 16.5 (C-25), 16.9 (C-26), 15.6 (C-27), 18.6 (C-28), 24.1 (C-29), 31.2 (C-30).

Compound **4**: ^1^H NMR (CDCl_3_, 400 MHz): *δ*_H_ 4.68 (1H, s, H_a_-20), 4.55 (1H, s, H_b_-20), 3.20 (1H, m, H-3), 1.67 (3H, s, H_3_-30), 1.02 (3H, s, H_3_-27), 0.95 (3H, s, H_3_-24), 0.86 (3H, s, H_3_-25), 0.82 (3H, s, H_3_-23), 0.78 (3H, s, H_3_-28), 0.75 (3H, s, H_3_-26); ^13^C NMR (CDCl_3_, 125 MHz): *δ*_C_ 37.3 (C-1), 29.8 (C-2), 79.1 (C-3), 40.1 (C-4), 55.4 (C-5), 18.4 (C-6), 34.4 (C-7), 42.9 (C-8), 50.6 (C-9), 38.2 (C-10), 21.0 (C-1l), 27.6 (C-12), 35.7 (C-13), 43.1 (C-14), 28.1 (C-15), 42.9 (C-16), 48.0 (C-17), 27.5 (C-18), 48.4 (C-19), 151.0 (C-20), 34.4 (C-21), 40.9 (C-22), 30.0 (C-23), 16.1 (C-24), 18.1 (C-25), 16.2 (C-26), 14.7 (C-27), 25.2 (C-28), 109.3 (C-29), 21.0 (C-30).

Compound **5**: ^1^H NMR (CDCl_3_, 600 MHz): *δ*_H_ 1.23 (3H, s, H_3_-29), 1.12 (3H, s, H_3_-30), 1.09 (3H, s, H_3_-25), 1.07 (3H, s, H_3_-26), 1.03 (3H, s, H_3_-27), 0.97 (3H, s, H_3_-24), 0.94 (3H, s, H_3_-23), 0.82 (3H, s, H_3_-28); ^13^C NMR (CDCl_3_, 125 MHz): *δ*_C_ 39.7 (C-1), 34.3 (C-2), 218.3 (C-3), 47.5 (C-4), 55.0 (C-5), 19.9 (C-6), 34.0 (C-7), 41.4 (C-8), 50.1 (C-9), 36.9 (C-10), 22.1 (C-11), 28.9 (C-12), 37.7 (C-13), 43.7 (C-14), 27.7 (C-15), 35.7 (C-16), 44.8 (C-17), 48.4 (C-18), 49.8 (C-19), 73.6 (C-20), 29.2 (C-21), 40.3 (C-22), 26.8 (C-23), 21.2 (C-24), 16.2 (C-25, C-26), 14.9 (C-27), 19.4 (C-28), 31.8 (C-29), 24.9 (C-30).

Compound **6**: ^1^H NMR (CD_3_OD, 400 MHz): *δ*_H_ 4.71 (1H, s, H_a_-29), 4.59 (1H, s, H_b_-29), 1.70 (3H, s, H_3_-30), 1.44 (3H, s, H_3_-23), 1.06 (3H, s, H_3_-24), 1.02 (3H, s, H_3_-26), 1.01 (3H, s, H_3_-27), 0.95 (3H, s, H_3_-25); ^13^C NMR (CD_3_OD, 101 MHz): *δ*_C_ 40.7 (C-1), 35.0 (C-2), 218.5 (C-3), 48.5 (C-4), 56.1 (C-5), 20.8 (C-6), 34.8 (C-7), 41.8 (C-8), 51.2 (C-9), 38.1 (C-10), 22.6 (C-11), 26.9 (C-12), 39.8 (C-13), 43.7 (C-14), 31.7 (C-15), 33.3 (C-16), 57.5 (C-17), 50.4 (C-18), 49.3 (C-19), 152.0 (C-20), 30.8 (C-21), 38.1 (C-22), 27.2 (C-23), 21.4 (C-24), 16.5 (C-25), 16.4 (C-26), 15.0 (C-27), 180.0 (C-28), 110.2 (C-29), 19.5 (C-30).

Compound **7**: ^1^H NMR (CDCl_3_, 400 MHz): *δ*_H_ 5.30 (1H, d, *J* = 7.0 Hz, H-21), 3.67 (1H, d, *J* = 11.0 Hz, H_a_-28), 3.48 (1H, d, *J* = 11.0 Hz, H_b_-28), 3.40 (1H, t, *J* = 2.8 Hz, H-3), 1.65 (3H, s, H_3_-30), 1.03 (3H, s, H_3_-25), 1.00 (3H, d, *J* = 6.5 Hz, H_3_-29), 0.99 (3H, s, H_3_-24), 0.94 (3H, s, H_3_-27), 0.86 (3H, s, H_3_-23), 0.83 (3H, s, H_3_-26); ^13^C NMR (CDCl_3_, 125 MHz): *δ*_C_ 33.5 (C-1), 25.5 (C-2), 76.4 (C-3), 37.7 (C-4), 48.8 (C-5), 18.4 (C-6), 34.2 (C-7), 41.5 (C-8), 50.3 (C-9), 37.4 (C-10), 21.7 (C-11), 26.8 (C-12), 38.4 (C-13), 42.4 (C-14), 27.7 (C-15), 30.3 (C-16), 38.8 (C-17), 49.2 (C-18), 36.5 (C-19), 141.2 (C-20), 118.0 (C-21), 35.2 (C-22), 28.4 (C-23), 23.1 (C-24), 16.2 (C-25), 16.2 (C-26), 15.1 (C-27), 60.4 (C-28), 22.3 (C-29), 21.5 (C-30).

Compound **8**: ^1^H NMR (CDCl_3_, 500 MHz): *δ*_H_ 5.12 (1H, t, *J* = 6.5 Hz, H-24), 4.48 (1H, dd, *J* = 10.6, 5.7 Hz, H-3), 2.04 (3H, s, CO*CH_3_*), 1.69 (3H, s, H_3_-26), 1.62 (3H, s, H_3_-27), 1.14 (3H, s, H_3_-21), 0.96 (3H, s, H_3_-18), 0.87 (3H, s, H_3_-28), 0.85 (9H, s, H_3_-19, H_3_-29, H_3_-30); ^13^C NMR (CDCl_3_, 125 MHz): *δ*_C_ 38.9 (C-1), 23.9 (C-2), 81.1 (C-3), 38.1 (C-4), 56.1 (C-5), 18.3 (C-6), 35.3 (C-7), 40.6 (C-8), 50.7 (C-9), 37.2 (C-10), 21.7 (C-11), 27.7 (C-12), 42.4 (C-13), 50.4 (C-14), 31.3 (C-15), 25.0 (C-16), 50.0 (C-17), 15.7 (C-18), 16.6 (C-19), 75.5 (C-20), 25.6 (C-21), 40.7 (C-22), 22.7 (C-23), 124.9 (C-24), 131.7 (C-25), 25.9 (C-26), 17.8 (C-27), 28.1 (C-28), 16.6 (C-29), 16.4 (C-30), 21.4 (CO*CH_3_*), 171.1 (*CO*CH_3_).

Compound **9**: ^1^H NMR (CDCl_3_, 400 MHz): *δ*_H_ 5.70 (1H, s, H-4), 1.16 (3H, s, H_3_-19), 0.99 (3H, d, *J* = 6.6 Hz, H_3_-21), 0.87 (3H, d, *J* = 7.0 Hz, H_3_-26), 0.82 (3H, t, *J* = 7.4 Hz, H_3_-29), 0.79 (3H, d, *J* = 7. 0 Hz, H_3_-27), 0.70 (3H, s, H_3_-18); ^13^C NMR (CDCl_3_, 125 MHz): *δ*_C_ 35.7 (C-1), 34.1 (C-2), 199.6 (C-3), 123.8 (C-4), 171.7 (C-5), 33.0 (C-6), 32.1 (C-7), 35.7 (C-8), 53.9 (C-9), 38.7 (C-10), 21.1 (C-11), 39.7 (C-12), 42.5 (C-13), 56.1 (C-14), 26.2 (C-15), 24.3 (C-16), 55.9 (C-17), 12.0 (C-18), 19.1 (C-19), 36.0 (C-20), 36.2 (C-21), 34.0 (C-22), 26.2 (C-23), 45.9 (C-24), 29.3 (C-25), 21.1 (C-26), 21.1 (C-27), 23.1 (C-28), 12.1 (C-29).

Compound **10**: ^1^H NMR (CDCl_3_, 400 MHz): *δ*_H_ 5.34 (1H, d, *J* = 5.1 Hz, H-6), 5.14 (1H, dd, *J* = 15.2, 8.6 Hz, H-22), 5.00 (1H, dd, *J* = 15.5, 8.6 Hz, H-23), 3.51 (1H, m, H-3), 1.00 (3H, s, H_3_-19), 0.91 (3H, d, *J* = 6.6 Hz, H_3_-21), 0.83 (3H, d, *J* = 7.2 Hz, H_3_-26), 0.81 (3H, d, *J* = 7.0 Hz, H_3_-27), 0.80 (3H, t, *J* = 7.0 Hz, H_3_-29), 0.67 (3H, s, H_3_-18); ^13^C NMR (CDCl_3_, 151 MHz): *δ*_C_ 37.0 (C-1), 31.3 (C-2), 71.5 (C-3), 41.4 (C-4), 141.2 (C-5), 121.3 (C-6), 34.6 (C-7), 31.5 (C-8), 50.6 (C-9), 36.8 (C-10), 21.4 (C-11), 23.4 (C-12), 42.0 (C-13), 56.6 (C-14), 24.2 (C-15), 28.0 (C-16), 56.7 (C-17), 12.4 (C-18), 19.2 (C-19), 36.0 (C-20), 18.7 (C-21), 139.0 (C-22), 129.2 (C-23), 45.7 (C-24), 29.8 (C-25), 20.3 (C-26), 19.4 (C-27), 26.6 (C-28), 11.7 (C-29).

### 3.5. In Vitro α-Glucosidase Inhibitory Activity Assay

α-Glucosidase activity was assessed according to a previous report [32]. α-Glucosidase (Sigma, G5003, St. Louis, MO, USA) derived from baker’s yeast and *p*-NPG (Sigma, N1377, Louis, MO, USA) as the substrate were purchased from Sigma-Aldrich. Acarbose (Shanghai Yuanye Bio-Technology Co., Ltd., Shanghai, China) was used as the positive control. The tested compounds and positive control acarbose were dissolved in DMSO (Shanghai Chemical Reagents Co., Ltd., Shanghai, China), and the enzyme and the substrate were dissolved in phosphate buffer with pH 6.86. The inhibitors and enzyme were pre-incubated in phosphate buffer at 37 °C for 15 min, and then 25 μL of substrate was added to the system to start the reaction, and the incubation was continued at 37 °C for 15 min. Finally, the reaction was terminated by the addition of 50 μL of 0.2 M reaction termination solution (Na_2_CO_3_, Shanghai Chemical Reagents Co., Ltd., Shanghai, China). The optical density (OD) was measured at an absorbance wavelength of 405 nm using a microplate reader (Thermo Fisher Scientific, Waltham, MA, USA). The IC_50_ values were estimated with six different inhibitory concentrations, and each sample was measured three times in parallel experiments.

### 3.6. Molecular Docking

The crystal structure of *α*-glucosidase was downloaded from the Protein Data Bank (PDB ID: 3TOP, https://www.rcsb.org/, accessed on 1 September 2023). ADT 1.5.7 software was used to investigate the activity in terms of binding affinity (Kcal/mol), and thereafter, the outcomes were compared to the binding affinity score for best-docked conformation. The structures of compounds **1**, **2**, **4**, and **6** were drawn by ChemDraw 20.0 software and further converted to the 3D structure using Chem3D 20.0 software. The structure was optimized by energy minimization using the MM2 method and converted to a readable format at the ADT interface. The 3TOP receptor was imported into the software Pymol 2.4, and the GLC and AC1 groups contained in the 3TOP receptor file could be deleted; in addition, the water molecules could also be deleted in ADT or Pymol, and the pdb file was finally exported. The resulting files were imported into ADT, hydrated, merged with nonpolar hydrogen atoms, and saved as 3TOP.pdbqt. AutoDock Tools 1.5.7 offers two docking modes, semiflexible or rigid, and for this experiment, semiflexible docking was used. The ligand was imported into ADT and hydrogenated to convert it into a ligand. The ligand subroutine in AutoDock Tools 1.5.7 was used to identify the number of rotatable bonds that could rotate to dock with the receptor molecule during docking. The software detected the active site of 3TOP, and the coordinates were set to (−52.213 8.864 −64.710), while the docking parameter lattice spacing was set to 0.375 Å. The results were analyzed using Discovery Studio Visualizer 19.1.0 software, which revealed the presence of close-contact hydrogen bond interactions.

## 4. Conclusions

In summary, a detailed chemical investigation of the Chinese endemic plant *W. filipes* var. *tomentosum* led to the identification of an array of structurally diverse compounds, including two oleane-type triterpenes (**1** and **2**), four lupane-type triterpenes (**3**–**6**), one taraxastane-type triterpene (**7**), one dammarane-type triterpene (**8**), and two steroids (**9** and **10**). These components might serve as evidence of the close taxonomic relationship between the species *W. filipes* and its variant *W. filipes* var. *tomentosum* from the chemical perspective. All the isolates were assessed for the α-glucosidase inhibitory activity. As a result, compounds **1**, **2**, **4**, and **6** exhibited significant inhibition against α-glucosidase (IC_50_ values ranging from 16.6 to 19.2 μM), which were close in value to the positive control acarbose (IC_50_ 11.5 μM). This bioassay indicated these four compounds were potent α-glucosidase inhibitors, which could be developed as lead compounds against diabetes. The discovery of secondary metabolites of different types as well as the investigation of their bioactivities enriched the phytochemical and pharmacological diversities of *W. filipes*. Further studies on chemical modifications of these chemical constituents and the detailed mechanism of α-glucosidase inhibition are planned.

## Figures and Tables

**Table 1:** The results of α-glucosidase inhibitory bioassay of compounds **1**–**10** and positive control acarbose.

Compound	IC_50_ (μM)
**1**	16.8 ± 0.23
**2**	16.6 ± 0.25
**3**	<100
**4**	19.2 ± 0.17
**5**	<100
**6**	17.2 ± 0.27
**7**	<100
**8**	<100
**9**	<100
**10**	<100
acarbose	11.5 ± 0.05

## Data Availability

Data is contained within the article or Supplementary Material.

## Supplementary Materials

The following supporting information can be downloaded at: https://www.mdpi.com/article/10.3390/plants13050692/s1, Figure S1: The dose–response curves for compounds **1**–**10** and acarbose; Table S1: The α-glucosidase inhibition rates (%) at different concentrations for compounds **1**–**10** and acarbose.

## Sample Availability

Samples of the compounds are not available from the authors.
