# Supplementary material for: α-Glucosidase Inhibitory Phytochemical Components of Chinese Endemic Plant Whitfordiodendron filipes var. tomentosum
Source: Plants (Basel). 2024 Feb 29;13(5):692. doi: 10.3390/plants13050692 (PMC10934055; doi:10.3390/plants13050692)
Supplement: Supplementary file 1 [file plants-13-00692-s001.zip › plants-2821872-supplementary.pdf]

Supplementary Materials

α-Glucosidase Inhibitory Phytochemical Components of Chinese Endemic Plant *Whitfordiodendron filipes* var. *tomentosum*

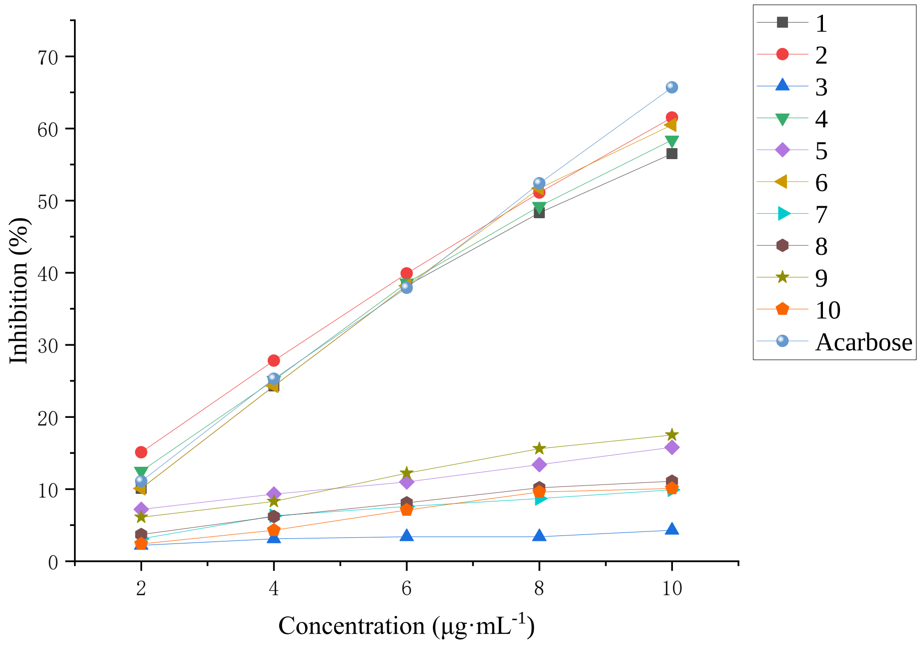

Figure S1. The dose-response curves for compounds **1**–**10** and acarbose.

Table S1. The α-glucosidase inhibition rates (%) at different concentrations for compounds **1–10** and acarbose.

Concentration (μg·mL^{-1})	**1**	**2**	**3**	**4**	**5**	**6**	**8**	**7**	**9**	**10**	Acarbose
2.0	10.1	15.1	2.2	12.5	7.2	10.1	3.7	3.1	6.1	2.4	11.1
4.0	24.3	27.8	3.1	25.1	9.3	24.3	6.2	6.3	8.3	4.3	25.3
6.0	38.2	39.9	3.4	38.6	11.0	38.2	8.1	7.6	12.2	7.1	37.9
8.0	48.3	51.1	3.4	49.2	13.4	51.7	10.2	8.7	15.6	9.6	52.4
10.0	56.5	61.5	4.3	58.4	15.8	60.5	11.1	9.9	17.5	10.1	65.7
